# A Feasibility Study on Conducting Elective Surgeries During Weekends and Evening Hours in a Public Hospital in Riyadh, Saudi Arabia

**DOI:** 10.7759/cureus.87824

**Published:** 2025-07-13

**Authors:** Mamdouh Falih Althaqeel, Abdulaziz Serhan Alkhaldi, Mohammed Nawras Alshanawani, Khalid J Alqahtani, Arwa Hussain Aljohani, Mohammad Shibly Khan, Walid Abdullah Altasaan, Saud Ibrahim Binjudiaan

**Affiliations:** 1 Hospital Administration, King Salman Hospital, Riyadh, SAU; 2 Hospital Administration, Al-Iman General Hospital, Riyadh, SAU; 3 Surgery, King Salman Hospital, Riyadh, SAU; 4 Pediatric Surgery, King Salman Hospital, Riyadh, SAU; 5 Health Excellence, King Salman Hospital, Riyadh, SAU

**Keywords:** elective surgeries, hospital management, operating room performance, or scheduling, planned care, weekend surgeries

## Abstract

Background: The working schedule for operating rooms (ORs) in public hospitals in Saudi Arabia spans five days a week, with eight working hours per day. However, to meet the surgical needs of our beneficiaries, we extended the elective operating room schedule by adding extra slots during evenings and weekends.

Methods: This is a retrospective analysis of elective surgical cases conducted at King Salman Hospital, Riyadh, from January 2024 to December 2024. The cases were categorized into two main groups: those conducted during the routine weekday schedule and those performed during the extended list (weekends and evenings). Unplanned return to the operating room within 24 hours of the primary procedure and 30-day mortality were identified as the main outcome measures. Chi-square and Fisher's exact tests were applied to test the statistical significance between the two groups.

Results: During 2024, a total of 5024 routine surgeries were performed at our hospital, of which 30% (1550/5024) were conducted during extended hours: 21% (1080/5024) during weekends and 9% (470/5024) during evening hours. Of the total cases conducted during the extended list, about 78% (1206/1550) were day surgeries, while about 40% (625/1550) were major in complexity. The mortality rate for weekday elective surgeries was observed to be 0.12% (4/3474), while there were no deaths among weekend elective surgeries (p < 0.001). There was only one case of unplanned return to the OR, which occurred during the main weekday OR list.

Conclusion: Performing elective surgeries during nontraditional working hours is a safe and efficient approach to maximize hospital resources.

## Introduction

Operating room (OR) performance is a broad term that encompasses many aspects, ranging from efficiency, quality of care, patient safety, and utilization [1]. The scheduling of the OR is not only a major determinant of access to care in a health care system, but it also has a significant impact on the effective utilization of the operating room [2, 3]. Surgical services represent major areas of revenue in health services. The creation of self-operating accountable care organizations in the form of health clusters is one of the core health care transformation strategies in Saudi Arabia [4]. Also, timely provision of elective surgical services is another health-related goal, reflected in the model of care strategies under health care transformation in Saudi Arabia [4].

Health systems across the world are trying to restructure their five-day workweek model by providing elective surgical services on the weekends as well [5-7]. While some aim to reduce the elective surgical waiting list backlog through weekend surgeries [6], others have adopted weekday evening surgeries to expand services and improve access [7]. The disparity in the availability of resources during weekends and weekdays is a major constraint for not performing elective cases during weekends [8]. 

King Salman Hospital (KSH) is one of the leading secondary care public hospitals in the capital city Riyadh in Saudi Arabia, serving a population of more than one million residents. Surgical services are one of the most sought-after services in a secondary care hospital. Catering to a large catchment population, the surgery coordination office at King Salman Hospital had a significant waiting list with many cases pending for a long duration. While we had a large waiting list, new cases were also being added due to increased demand. After the resumption of services following the COVID-19 disruption phase, we experienced rapid pooling of the waiting list. The main factors responsible for the backlog of surgical cases were related to infrastructural capacity, with only three elective operating rooms at our hospital. Patient-related factors such as no-show rates and surgical cancellations also adversely affected OR efficiency. Considering the surgical needs of our beneficiaries and to improve access to surgical services, we implemented an extended surgical list schedule by adding extra slots to the operating room. The present study was conducted with the objective of comparing patient safety and clinical outcomes among elective surgical cases conducted during extended hours (weekend/evening list) and those conducted during regular weekday working hours.

## Materials and methods

Study design and settings

There are three elective operating rooms at our hospital, in addition to one operating room for emergency procedures and one delivery room for obstetrics cases. The elective operating rooms operate during 5 working days, from 8 AM to 4 PM, while the emergency room and delivery room are open 24 hours. All the major specialties are available at our hospital, along with a dedicated anesthesia department. After the mutual agreement of all the stakeholders, we extended the working hours of the operating rooms by creating extra slots in each of the three elective rooms during the evening time, starting from 4 PM to 11 PM, and during weekends (8 AM to 4 PM). In coordination with the departments of anesthesia, nursing, and supportive services, we drafted surgical schedules for the evening hours and for weekends. The staff (surgeon, anesthesia, nursing, etc.) working during those hours were arranged for their shifts accordingly, with flexibility in the off-hours during weekdays and weekends. To ensure patient safety, these shifts were scheduled to include only consultants for both surgical specialties and anesthesia. The operating surgeons were offered the option to plan their routine surgical cases on any day of the week. The evening and weekend shifts were allotted on a priority basis to the specialty with a high waiting list. Nevertheless, the surgeons were given the choice to perform routine surgery on a volunteer basis. The surgical coordination office was instructed to shortlist the cases with long waiting times and to contact them to assess their preferences for getting the surgeries done.

Data management

This study involved a retrospective analysis of routinely collected hospital data. Patient confidentiality was maintained throughout. The complete data set was prepared by the OR administration for each of the performed cases, as part of routine data collection. The data include basic information of the cases, diagnosis, procedure performed, complexity of the procedures, names of surgeons and anesthetists who performed and assisted, all the time stamps required in the surgical procedure, and the American Society of Anesthesiologists (ASA) levels. The manual data were collected and compiled on an Excel sheet (Microsoft Corporation, Redmond, Washington) and were analyzed using IBM SPSS Statistics for Windows, Version 21 (Released 2012; IBM Corp., Armonk, New York). Chi-square and Fisher's exact tests were used to find the association between the variables. A p-value of 0.05 was set to define statistical significance.

## Results

A total of 7220 surgical procedures were identified for the year 2024 at our hospital, comprising about 69.6% (5024/7220) routine cases and 29.9% (2196/7220) emergency procedures. The cases performed during the extended OR schedule contributed to about 31% (1550/5024), representing about 21% (1080/5024) conducted during weekends and 9% (470/5024) during evening hours.

Table 1 shows the comparison of patient characteristics between the main OR list and the extended list. While there was a significant difference between the main and extended list cases (p < 0.01), no significant difference was observed in patient outcomes (p > 0.05). During the extended list, about 58% of cases were conducted as day surgery, about 60% were major in complexity, and the majority (54%) belonged to ASA level 1. 

**Table 1:** Comparison of elective surgical procedures performed during the main OR list (weekdays 8:00 AM–4:00 PM) and extended list (weekends and evenings) (N = 5024) (*Chi-square test, **Fisher exact test) ASA: American Society of Anesthesiologists, OR: operating rooms.

Patient characteristics		Extended list	Main OR list	Total	p-value
Sex of the patients	Male	802 (51.7%)	1996 (57.5%)	2798 (55.7%)	ꭓ^2^=14.176, p<0.001*
	Female	748 (48.3%)	1478 (42.5%)	2226 (44.3%)	
Type of surgery	Day surgery	1206 (77.8%)	2025 (58.3%)	3231 (64.3%)	ꭓ^2=^177.864, p <0.001*
	Inpatient	344 (22.2%)	1449 (41.7%)	1793 (35.7%)	
Complexity of cases	Major	625 (40.3%)	2095 (60.3%)	2720 (54.1%)	ꭓ^2=^218.371, p <0.001*
	Minor	918 (59.2%)	1296 (37.3%)	2214 (44.1%)	
	Intermediate	7 (0.5%)	83 (2.4%)	90 (1.8%)	
ASA level	ASA 1	751 (48.5%)	1890 (54.4%)	2641 (52.6%	F=56.272, p <0.001**
	ASA 2	767 (49.5%)	1407 (40.5%)	2174 (43.3%)	
	ASA 3	29 (1.9%)	163 (4.7%)	192 (3.8%)	
	ASA 4	2 (0.1%)	14 (0.4%)	16 (0.3%)	
	ASA 5	1 (0.1%)	0 (0.0%)	1 (0.0%)	
30 Days mortality	Yes	0 (0%)	4 (0.1%)	4 (0.1%)	F=0.388, p 0.181**
	No	1550 (100%)	3474 (99.9%)	5020 (99.9%)	

The 30-day mortality was observed to be 0.1% among the cases conducted during the main list, while there was no mortality among the cases conducted during the weekend and evening lists. Among the cases performed in the main list, only one case had an unplanned return to the operating room within 24 hours of the primary procedure, while there were none among the cases conducted during the extended list.

Table 2 shows the distribution of cases performed according to specialties. Out of the total elective surgical cases, the highest volume in the extended list was contributed by Ophthalmology (48.2%, 747/1550), while General Surgery contributed the highest (25.5%, 887/3474) in the main OR list. Endoscopic procedures and Vascular Surgery were performed only during the regular operating room schedule. 

**Table 2:** Elective surgical volume according to the specialties during main OR list and extended OR list (chi-squared test) OR: operating rooms.

Speciality	Extended list		Main OR list		Total (% of sample)		p-value
Ophthalmology	747	48.2%	441	12.7%	1188	23.6%	ꭓ^2^=1015.196, p<0.001
General surgery	412	26.6%	887	25.5%	1299	25.9%	
Orthopaedic surgery	148	9.5%	464	13.4%	612	12.2%	
Bariatric	106	6.8%	190	5.5%	296	5.9%	
ENT	76	4.9%	412	11.9%	488	9.7%	
Gynecology	16	1.0%	63	1.8%	79	1.6%	
Urology	13	0.8%	425	12.2%	438	8.7%	
Neurosurgery	11	0.7%	24	0.7%	35	0.7%	
Obstetrics	8	0.5%	141	4.1%	149	3.0%	
Paediatric surgery	4	0.3%	233	6.7%	237	4.7%	
Plastic surgery	4	0.3%	122	3.5%	126	2.5%	
Dental surgery	3	0.2%	26	0.7%	29	0.6%	
Thoracic surgery	2	0.1%	3	0.1%	5	0.1%	
Vascular surgery	0	0.0%	33	0.9%	33	0.7%	
Endoscopy	0	0.0%	10	0.3%	10	0.2%	
Total	1550	100%	3474	100%	5024	100%	

## Discussion

In this study, we have demonstrated the usefulness and relevance of adopting non-traditional working hours for the operating room in a secondary care public hospital, through innovation in hospital administration policies. While we had a significant improvement in the overall surgical volume at our hospital, the surgeries conducted during extended hours contributed to about one-fifth of the total annual volume of elective surgeries. In a similar approach, Ong et al. introduced a concept of twilight operating room functioning, whereby they extended their OR working hours from 5 PM to 8:30 PM, along with a weekend list [9].

The impact of our project was observed across various aspects. Foremostly, we were able to utilize the available capacity and manpower more efficiently, which is evident not only in the improved surgical volume but also in sustaining the OR utilization rate. By creating standby OR schedules, we were able to mitigate the loss of OR hours due to cancellations and patient no-shows. Flexible OR scheduling has been reported to improve the efficiency of the OR in a tertiary care hospital in South Korea [10].

We were able to implement a culture change in our hospital among the surgical community. As a result, the surgeons are opting to perform more surgeries based on their preferred schedules. Since surgeons have the flexibility to schedule their cases according to their day and time preferences, the quality of the procedures performed has improved, along with patient safety. None of these cases had an unplanned return to the OR, and no major postoperative complications were observed, which demonstrates the assurance of patient safety. Our results are corroborated by another study conducted in Japan, which demonstrated no increase in mortality among elective cases conducted during weekends [5]. Similarly, a study conducted in Australia reported no major postoperative complications among elective cases conducted during evening hours [9]. In a systematic review, it was reported that cases conducted during extra hours were associated with a higher mortality rate compared to those conducted during daytime hours. However, this conclusion was based on both emergency and elective procedures [10].

The extended OR hour scheduling has been sustained for the past two years and is still in place at our hospital, which demonstrates the sustainability of our innovation. The continued cooperation of the concerned stakeholders is one of the most important cornerstones of this innovation to maintain it on a long-term basis. Dedicated staff assigned to evening and weekend operating rooms is one of the most effective strategies to sustain this innovation. Moreover, the overrunning of afternoon OR sessions was not an impeding factor for the evening sessions due to the separate staff available, thus ruling out delays in surgical start times, which have been reported to be a crucial factor affecting OR efficiency [11,12].

The health care transformation plan in Saudi Arabia has envisaged the delivery of health care services through the accountable care organization (ACO), materialized in the creation of a set of health clusters consisting of tertiary care and secondary care hospitals along with primary health centers [13]. Health care services are gradually moving towards privatization, whereby providers will have to generate their own resources through the provision of health services. Innovations such as the extended elective OR list pave the way for expanding revenue generation while utilizing the same infrastructure and resources.

## Conclusions

A non-traditional approach to surgical scheduling, such as weekend and evening elective surgeries, is an effective strategy to improve overall hospital performance, especially in the context of resource constraints. We found no adverse patient outcomes associated with performing elective surgeries during weekends and evenings. Needless to mention, it provides more freedom to the operating surgeon to plan their cases without being constrained by operating room availability.

Our innovation paves the way for realization of the objectives of healthcare transformation in Saudi Arabia, whereby healthcare entities, referred to as ACOs, will be responsible for their revenue generation while maintaining the quality of services and patient safety, along with ensuring optimum access to care. As surgical services are a major source of revenue generation, expanding these services is the way forward for healthcare entities.
